# Biorenewable triblock copolymers consisting of l-lactide and ε-caprolactone for removing organic pollutants from water: a lifecycle neutral solution

**DOI:** 10.1186/s13065-019-0638-z

**Published:** 2019-10-22

**Authors:** Katrina T. Bernhardt, Haley G. Collins, Amy M. Balija

**Affiliations:** 1 Department of Chemistry, Fordham University, 441 East Fordham Road, Bronx, NY 10458 USA; 2 Department of Chemistry, Radford University, P.O. Box 6949, Radford, VA 24142 USA

**Keywords:** Pollution remediation, Polycyclic aromatic hydrocarbons, Rose Bengal, Poly(lactic acid), Poly(ε-caprolactone), Biorenewable polymers

## Abstract

**Background:**

Current methods of removing organic pollutants from water are becoming ineffective as the world population increases. In this study, a series of biorenewable triblock copolymers with hydrophobic poly(ε-caprolactone) block and hydrophilic poly(l-lactide) blocks were synthesized and tested as agents to remove environmental pollutants from an aqueous solution. The percent of pollutant removed and equilibrium inclusion constants were calculated for the polymers. These values were compared to previously known removal agents for their effectiveness.

**Results:**

Triblock copolymer samples removed over 70% of the polycyclic aromatic hydrocarbon (PAH) phenanthrene from an aqueous solution, with selectivity for the adsorption of phenanthrene over other PAHs tested. The inclusion constant was 7.4 × 10^5^ M^−1^ and adsorption capacity was 5.8 × 10^−7^ mol phenanthrene/g polymer. Rose Bengal was used to further probe the nature of interactions between the copolymers and a small molecule guest. Solid samples of the *block*-poly(l-lactide)–*block*-poly(ε-caprolactone)–*block*-poly(l-lactide) (PLLA–PCL–PLLA) systems were found to rapidly remove over 90% of Rose Bengal from aqueous solution, resulting in a complete disappearance of the characteristic pink color. Solutions of the copolymers in dichloromethane also removed Rose Bengal from water with a similar level of efficiency. Large inclusion constant values were obtained, ranging from 1.0 × 10^5^ to 7.9 × 10^5^ M^−1^, and the average adsorption capacity value of 6.2 × 10^−7^ mol/g polymer was determined. Aged polymer samples exhibited different adsorption characteristics and mechanistic theories for the removal of Rose Bengal were determined.

**Conclusion:**

The triblock copolymer consisting of l-lactide and ε-caprolactone was effective in removing various organic pollutants in aqueous environments. It is a biorenewable material which leads to minimal waste production during its lifecycle. These polymers were in general more effective in removing organic pollutants than commercially available pollution removal systems.
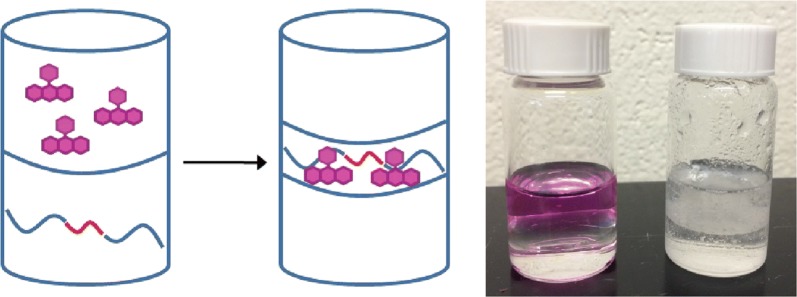

## Introduction

Providing potable water to a growing global population is complex due to the wide array of pollutants present in water supplies. Research has focused on removing metal ions [1–3], but drinking water contaminated with organic pollutants from industry, agriculture, pharmaceuticals, and personal care products is challenging. A new generation of materials that remove organic compounds from water is needed to address this unmet urgent need [4, 5]. Traditional remediation methods such as activated carbon sequester volatile organic compounds but are ineffective in removing non-volatiles, microplastics, and persistent organic pollutants (POPs) [6]. Cross-linked cyclodextrins, dendrimers, mineral clay nanoparticles, and hyper-branched polymers are an alternative approach to remove pollutants from aqueous matrices, although their high production costs, waste by-products, and degradation may be prohibitive for commercial applications [7–9].

One way to lower the cost of these materials is to utilize environmentally benign materials such as poly(lactide) [10–12]. Previous researchers have attached poly(lactide) to the surface of self-assembled porous films, metal-coordinating polymers, biomass hybrids, and enzyme-linked polymers [13–17]. The resulting polymers behave as nanosponges to remove and store the contamination. Langer exemplified this approach using poly(lactide) and poly(ethylene glycol) (PEG) copolymer nanoparticles [18]. Hydrophobic pollutants such as bis-phenol A (BPA), phthalates, and polycyclic aromatic hydrocarbons (PAHs) were removed from the aqueous environment by binding to the nanoparticle surface through the hydrophobic effect. Yet, the presence of hydrophilic PEG blocks may not provide optimal intermolecular interactions with hydrophobic organic pollutants.

**Fig. 1:**
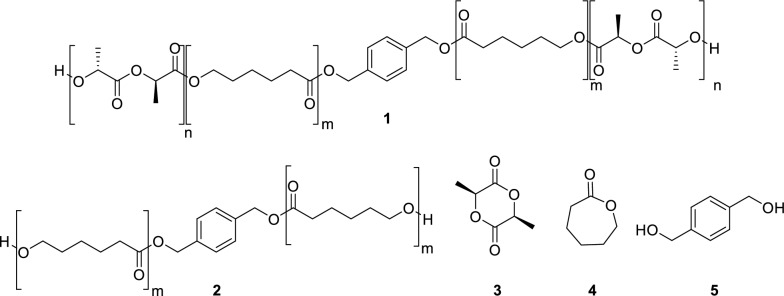
Structures of polymers **1** and **2** and starting materials **3**–**5**

Herein a unique approach for removing small organic compounds and PAHs from water using *block*-poly(l-lactide)–*block*-poly(ε-caprolactone)–*block*-poly(l-lactide) triblock copolymers **1** (PLLA–PCL–PLLA) is reported (Fig. 1). These block copolymers are prepared without starting material purification or stringent reaction conditions, unlike previous reported methods [19]. The hydrophobic poly(ε-caprolactone) chains are proposed to facilitate organic substrate adsorption while the hydrophilic poly(l-lactide) blocks will improve water compatibility. Combining poly(l-lactide) blocks and poly(ε-caprolactone) blocks is hypothesized to improve mechanical properties of the resulting system compared to each respective homopolymer [20].

Copolymers **1** were designed for sustainability with minimal waste products throughout the lifecycle of the polymers. Monomers **3** and **4** are readily available feedstock chemicals, and l-lactide (**5**) is derived from renewable resources. The synthesis of **1** proceeds through two efficient ring-opening polymerizations with l-lactide (**3**), ε-caprolactone (**4**), and non-toxic reagents [21, 22]. No reagents require purification prior to use, limiting energy consumption. The polymers are designed to be isolated through precipitation using methanol, a renewable resource [23]. At the end of its lifecycle, polymer **1** is proposed to degrade into environmentally benign l-lactic acid and ε-hydroxyhexanoic acid. Further degradation of ε-hydroxyhexanoic acid by microorganisms occurs through a β-oxidation mechanism to form carbon dioxide and water [24, 25]. Minimal pollution is expected from copolymers **1**, and their net environmental effects are negligible.

Given their potential as sustainable pollution remediation materials, a series of copolymers **1** were investigated. The molar ratio of poly(l-lactide) and poly(ε-caprolactone) blocks was varied to determine the optimal composition to remove effectively organic pollutants from water. The copolymers were tested in the removal of several PAHs from water and the organic dye Rose Bengal. The copolymer efficiency was compared with the commercially available Brita filters, poly(ethylene imine) (PEI), β-cyclodextrin, and 2nd generation poly(amidoamine) (PAMAM) dendrimers and shown to be superior.

## Results and discussion

### Polymer characterization

^1^H NMR spectroscopy was utilized to characterize the molecular weight of polymers **1a**–**1l**. Although end-group analysis is a standard method for determining M_n_, it was not possible in this circumstance because the ^1^H NMR peaks of these end group protons were not clearly resolved from other resonances [22, 26]. The relative integration of three ^1^H NMR signals corresponding to the poly(l-lactide), poly(ε-caprolactone), and 1,4-benzene dimethanol blocks were utilized to determine the experimental M_n_ (Table 1). Ranging from 1.7 × 10^4^ to 3.5 × 10^4^ Da, these M_n_ values were close in magnitude to the molar masses expected from complete polymerization. Polymer purity, M_n_, M_w_, and polydispersity index (PDI) values were determined by SEC to verify further the formation of polymer **1**. The PDI values were low, ranging from 1.03 to 1.05 and were consistent, indicating the formation of relatively homogeneous polymers (Table 1). A linear correlation between l-lactide: ε-caprolactone and M_n_ was observed. The percent relative standard deviations of the M_n_ values ranged from 0.3 to 14%, indicating acceptable reproducibility.

**Table 1:** Polymer composition variations based upon the amount of **3** added during synthesis, the ratio of **3** to **4**, and the molecular weight based on ^1^H NMR relative peak integrations

Polymer	Mole ratio 3:4	Average % yield^a^	Average E factor^a^	Average ^1^H NMR M_n_ (Da)^a^	Standard deviation^b^	% RSD^b^	Polydispersity (PDI)
**1a**	0.056	77	35	1.1 × 10^4^	4.0 × 10^1^	0.40	1.03
**1b**	0.11	71	36	1.1 × 10^4^	1.5 × 10^3^	14	1.03
**1c**	0.17	83	28	1.2 × 10^4^	1.1 × 10^3^	9.3	1.04
**1d**	0.23	76	30	1.2 × 10^4^	4.2 × 10^2^	3.6	1.03
**1e**	0.28	79	27	1.3 × 10^4^	4.0 × 10^1^	0.30	1.03
**1f**	0.34	81	25	1.3 × 10^4^	3.7 × 10^2^	2.7	1.04
**1g**	0.45	77	24	1.8 × 10^4^	1.6 × 10^3^	9.2	1.04
**1h**	0.56	73	22	1.9 × 10^4^	6.1 × 10^2^	3.3	1.04
**1i**	0.68	79	20	2.0 × 10^4^	8.5 × 10^2^	4.2	1.05
**1j**	0.79	77	19	2.3 × 10^4^	1.9 × 10^3^	8.3	1.05
**1k**	0.90	76	18	2.4 × 10^4^	8.6 × 10^2^	3.6	1.04
**1l**	0.96	68	22	2.5 × 10^4^	1.6 × 10^3^	6.1	1.05
**2**	0.00	N.D.	N.D.	9.7 × 10^3^	1.3 × 10^3^	13	1.05

% RSD is the percent relative standard deviation

^a^Averaged over duplicate or triplicate synthesis trials

^b^With respect to average ^1^H NMR M_n_

### Scanning electron microscopy (SEM)

**Fig. 2:**
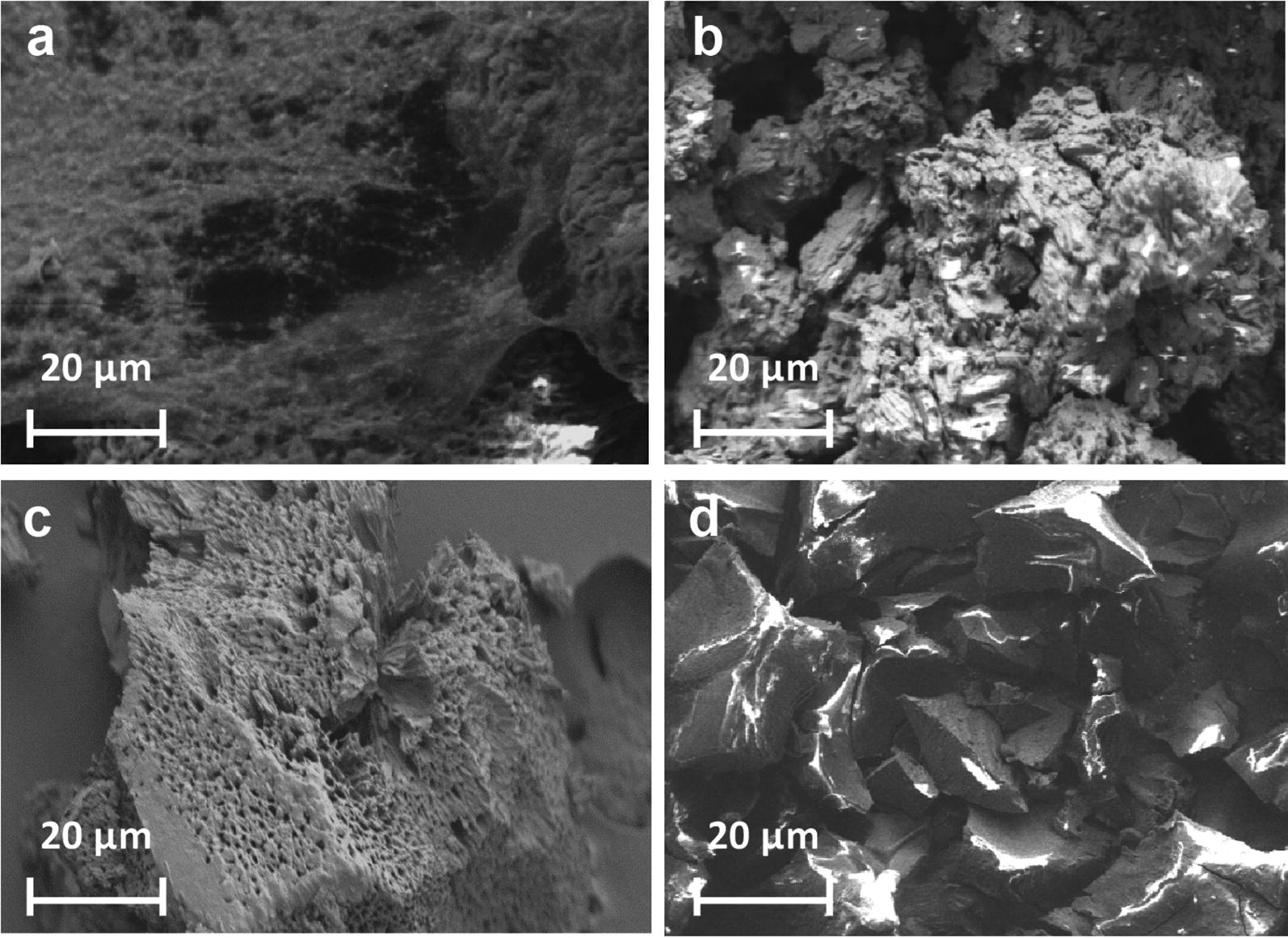
SEM images of polymers **a 1c**, **b 1g**, **c 1i** and **d 1l**

Polymers **1c**, **1g**, **1i**, and **1l**, were studied by SEM to examine how increasing the l-lactide block impacts morphology. The polymer surface changed from being a solid with high surface areas containing numerous crevices (i.e. **1c** and **1g**) to a material that has a smoother surface with a limited number of crevices (i.e. **1i** and **1l**) as the amount of l-lactide increased (Fig. 2) [20]. Previous research indicated that larger surface areas lead to more effective adsorption of organic compounds on nanosponges [1]. The differing solid-state morphologies with polymer **1** is proposed to impact the ability of the polymers to remove organic pollutants.

### Green chemistry principles

The synthesis of **1a**–**1l** follows atom economy and green chemistry principles since no stoichiometric waste products are produced, minimal organic solvents are employed, and the tin(II) octoate catalyst is non-toxic [27, 28]. To quantify waste formation, *E* factors were determined for each polymer [29]. Average *E* factors for polymers **1a**–**1l** (Table 1) ranged from 18 to 36. These *E* factors compare favorably with other laboratory scale processes, although additional optimization may be needed for large scale manufacturing. The methanol wash was the most significant waste product. In a commercial setting, methanol can be recovered and reused. Under that assumption, the overall *E* factors drop to 0.3–1.7, which are comparable to *E* factors for specialty chemical manufacturing processes and are more economically feasible.

The polymer synthesis is cost effective. Polymer **1** is derived from relatively inexpensive monomers: ε-caprolactone (**4**) costs $0.21 (US) per gram while l-lactide (**3**) costs $2.40 (US) per gram [30]. The initiator, 1,4-benzenedimethanol (**5**), is derived from the industrial feedstock terephthalic acid and is used in small quantities while the catalyst tin (II) octoate is a readily available reagent priced at $0.16 (US) per gram. The low cost of reagents indicate that a pollution remediation based on the copolymer would be commercially viable.

### Removal of polycyclic aromatic hydrocarbons (PAHs) from water

Polycyclic aromatic hydrocarbons (PAHs) are known persistent organic pollutants and suspected carcinogens that increasingly are being detected in water supplies [31]. Typical methods to remove PAHs from water involve activated carbon or activated carbon combined with coagulants although they are not 100% effective [32]. These pollutants were employed to examine polymer **1** as a sustainable solid-state pollution remediation device. Solid polymer **1l** was treated with saturated aqueous solutions of three PAHs: pyrene (**6**), fluoranthene (**7**), and phenanthrene (**8**). As a control, saturated aqueous solutions of **6**–**8** were treated with homopolymer **2**.

**Fig. 3:**
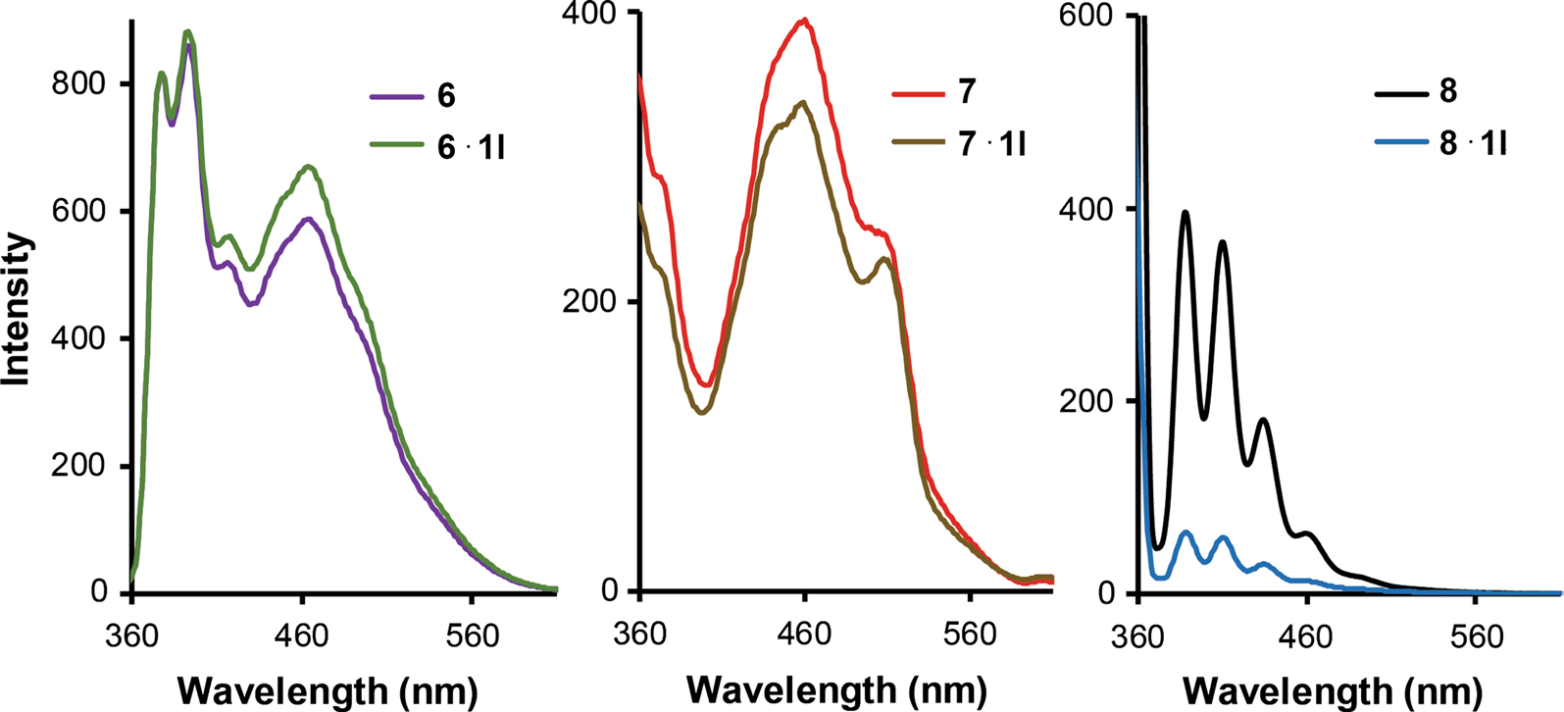
Comparison of fluorescence data obtained from three PAH solutions in water before and after the addition of triblock copolymers **1l**

The percent removal of PAH from an aqueous solution was determined by subtracting the fluorescence intensity of the analyte before and after exposure to the solid polymer (Table 2). A decrease in fluorescence intensity after exposure to the polymer was hypothesized to be PAH removal (Fig. 3). The block copolymers selectively removed PAHs from solution. After exposure to polymer **1l**, 70% of phenanthrene was removed from the aqueous environment while only 15% of fluoranthene and 4% of pyrene were removed after 30 s. In the case of pyrene, the excimer was observed under the experimental conditions both in the absence and presence of the polymer [33]. Phenanthrene was removed selectively over the other PAHs, even though they had similar structures. Yet, when the same PAHs were exposed to homopolymer **2**, an average 67% fluorescence decrease of fluoranthene and phenanthrene and a 19% fluorescence decrease of pyrene was observed. The results suggest that the poly(l-lactide) block selectively impacts PAH removal. The reason for this is unclear although the selectivity for PAHs may be influenced by the three-dimensional structure adopted by the poly(l-lactide) blocks.

**Table 2:** Comparison of % removal of PAHs from a saturated aqueous solution by the homopolymer **2** and the triblock copolymer **1l**

Material	% removal pyrene	% removal fluoranthene	% removal phenanthrene
**1l**	4.0	15	71
**2**	19	59	74
Brita filter	21	38	39

These results were compared with a Brita filter application which consists of activated charcoal and an ion resin. As shown in Table 2, **1l** was slightly less effective than the Brita filter in removing pyrene and fluoranthene but more effective in removing phenanthrene. Homopolymer **2** in general could remove all the PAHs from the aqueous solution. These results indicate that the polymer overall is more effective in removing the organic pollutants compared with the Brita filter which contains two different remediation materials. Other commercially available known adsorption materials such as PEI, 2nd generation PAMAM dendrimers, and β-cyclodextrin could not be examined due to water solubility or spectroscopic complications [34]. Biorenewable polymers **1** removed fluoranthene from an aqueous environment at comparable percentages as PEI, β-cyclodextrin, and diaminobutane poly(propylene imine) dendrimers impregnated on TiO_2_ filters (70–80% in 30 s) [35, 36]. All polymers tested were less effective in removing pyrene than the other PAHs from water.

To examine the polymer adsorptivity effectiveness, the inclusion constants and adsorption capacities were calculated for **1l** with the three PAHs (Table 3). The inclusion constant values (K_*i*_) are large, ranging from 4.4 × 10^5^ to 7.4 × 10^5^ M^−1^, indicating that PAH removal from water by the polymer is a thermodynamically favorable process [37].

**Table 3:** Inclusion constants and adsorption capacity values calculated for the removal of PAHs from a saturated aqueous solution by triblock copolymer **1l**

PAH	*K*_*i*_ (M^−1^)	Adsorption capacity (mol PAH/g polymer)
Pyrene	4.9 × 10^5^	5.4 × 10^−8^
Fluoranthene	4.4 × 10^5^	2.5 × 10^−8^
Phenanthrene	7.4 × 10^5^	5.8 × 10^−7^

The adsorption capacity values in Table 3 quantitatively follow the percent PAH removal trend highlighted in Table 2. The polymer adsorbs a higher amount of phenanthrene over fluoranthene and pyrene. This trend was independently confirmed using binding isotherm experiments for each PAH (see Additional file 1). The K_*i*_ value and adsorption capacity of **1l** with pyrene suggest a more favorable interaction between pyrene and the polymer than is indicated by the 4% removal measurement. The later value is most likely affected by the reduced pyrene solubility in water relative to phenanthrene and fluoranthene [38]. The adsorption capacity values reported for phenanthrene and fluoranthene are lower limits as the binding isotherms for these two PAHs indicate that saturation binding was not fully achieved in the concentration range studied.

The K_*i*_ values were compared to other macromolecular systems, including alkylated β-cyclodextrin, hyperbranched poly(propylene imine) systems, and benzyl amine dendrimers (Table 4) [7, 35–37, 39]. While these K_*i*_ values are 10^3^ to 10^5^ fold greater, the difference likely results from specific functionalities such as the cyclodextrin or amine groups that are absent from polymer **1**. Although the inclusion constants for **1** are smaller than previously published systems, these polymers have distinct advantages including the ease of preparation, the use of non-toxic reagents, and an environmentally benign degradation pathway.

**Table 4:** Comparison of inclusion constants for triblock copolymers and other sorbent materials

Adsorbent material	Guest compounds	*K*_*i*_ (M^−1^)	Adsorption capacity (mol PAH/g polymer)
**1l**	Pyrene, fluoranthene, phenanthrene	4.4 × 10^5^–7.4 × 10^5^	5.4 × 10^−8^–5.8 × 10^−7^
Alkylated β-cyclodextrin	Phenanthrene	1.2 × 10^6^–1.8 × 10^6^	2.4 × 10^−5^–2.5 × 10^−5^
Alkylated poly(ethyleneimine)	Pyrene, fluoranthene, phenanthrene	1.0 × 10^7^–2.0 × 10^8^	5.4 × 10^−5^–1.0 × 10^−4^
Alkylated poly-(propyleneimine) dendrimers	Pyrene, fluoranthene, phenanthrene	1.5 × 10^6^–1.0 × 10^8^	3.0 × 10^−5^–3.8 × 10^−4^
Benzyl amine dendrimers	Pyrene	6.1 × 10^9^–1.6 × 10^11^	6.3 × 10^−8^–3.1 × 10^−7^

### Removal of Rose Bengal from water

To examine this phenomenon further, studies were performed with a reporter organic pollutant. Rose Bengal (**9**), a water-soluble organic dye and measurable environmental pollutant, was employed in solution-phase complexation studies because its spectrophotometric properties change in different environments [40]. Previous researchers have utilized Rose Bengal for its ease in examining the microenvironment of the dye using standard analytical techniques [41–45]. Removal of bromophenol blue, a sulfonic acid dye, from water was attempted but no appreciable amount of dye was removed by polymer **1**. The chemical and physical properties of Rose Bengal and bromophenol blue are different under the experimental conditions, leading to a difference in how they interact with the polymers [46].

**Fig. 4:**
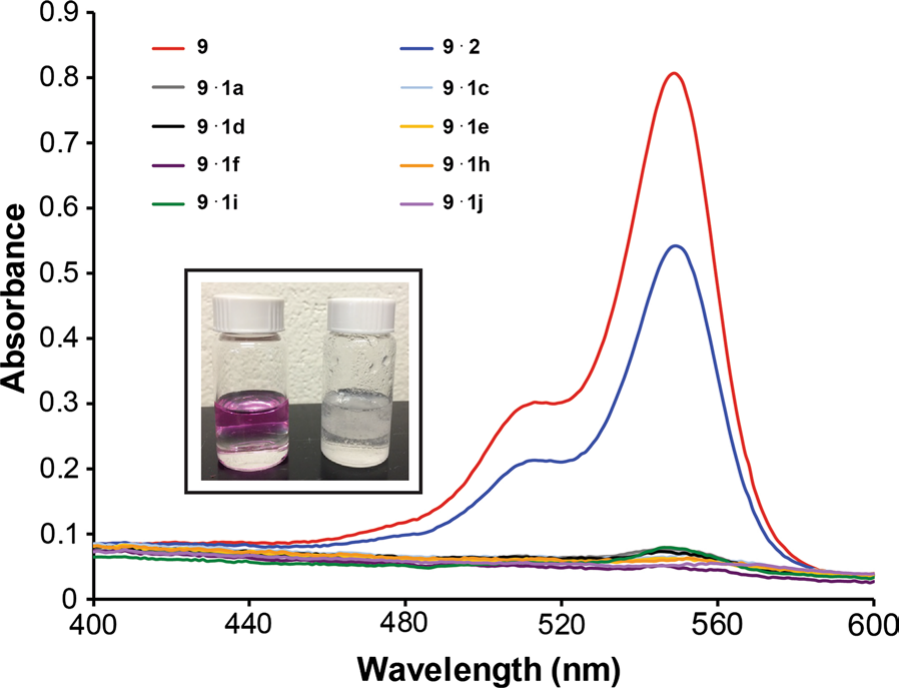
Overlay of UV/Vis spectra obtained during liquid–liquid extraction studies of select polymers **1** and 8.3 × 10^−6^ M Rose Bengal. The Rose Bengal absorbance decreased when combined with the polymers indicated in the figure legend. Inset: Comparison of aqueous (top layer) and organic (bottom layer) phases before mixing (left vial) and after mixing (right vial) with **1**. The characteristic pink color of Rose Bengal disappeared

An aqueous solution of Rose Bengal was mixed with a dichloromethane solution of the polymer for 30 s and analyzed by UV/Vis spectroscopy. A substantial decrease in the Rose Bengal absorbance was observed, indicating nearly complete removal of **9** from the aqueous phase (Fig. 4). Visually, the aqueous layer lost the characteristic pink color of Rose Bengal. Polymers **1a**–**1l** removed 85% Rose Bengal within 30 s of mixing, irrespective of the **3** to **4** ratio (Table 5). Homopolymer **2** was ineffective in removing the dye from water, with a removal average of 8.9%. These results suggest that while poly(l-lactide) is necessary for extracting Rose Bengal from aqueous solution, its block size does not impact significantly the percent dye removed.

**Table 5:** Comparison of % removal of Rose Bengal and calculated inclusion constants for the removal of Rose Bengal from water using the polymers

Polymer	% removal Rose Bengal at equilibrium	*K*_*i*_ (M^−1^)	∆G° (kcal/mol)
**2**	8.9	1.0 × 10^5^	− 6.8
**1d**	87	6.0 × 10^5^	− 7.8
**1f**	91	7.9 × 10^5^	− 8.0

No Rose Bengal UV/Vis absorption was detected in the organic or aqueous phases following extraction with polymer **1**. A pale pink polymer-like film formed at the interface between the aqueous and organic layers. This film, analyzed by ^1^H NMR spectroscopy, was consistent with the triblock copolymer. While there was visual evidence the Rose Bengal complexed with the polymer, no spectroscopic evidence by ^1^H NMR spectroscopy could be obtained due to the low concentration of Rose Bengal.

**Fig. 5:**
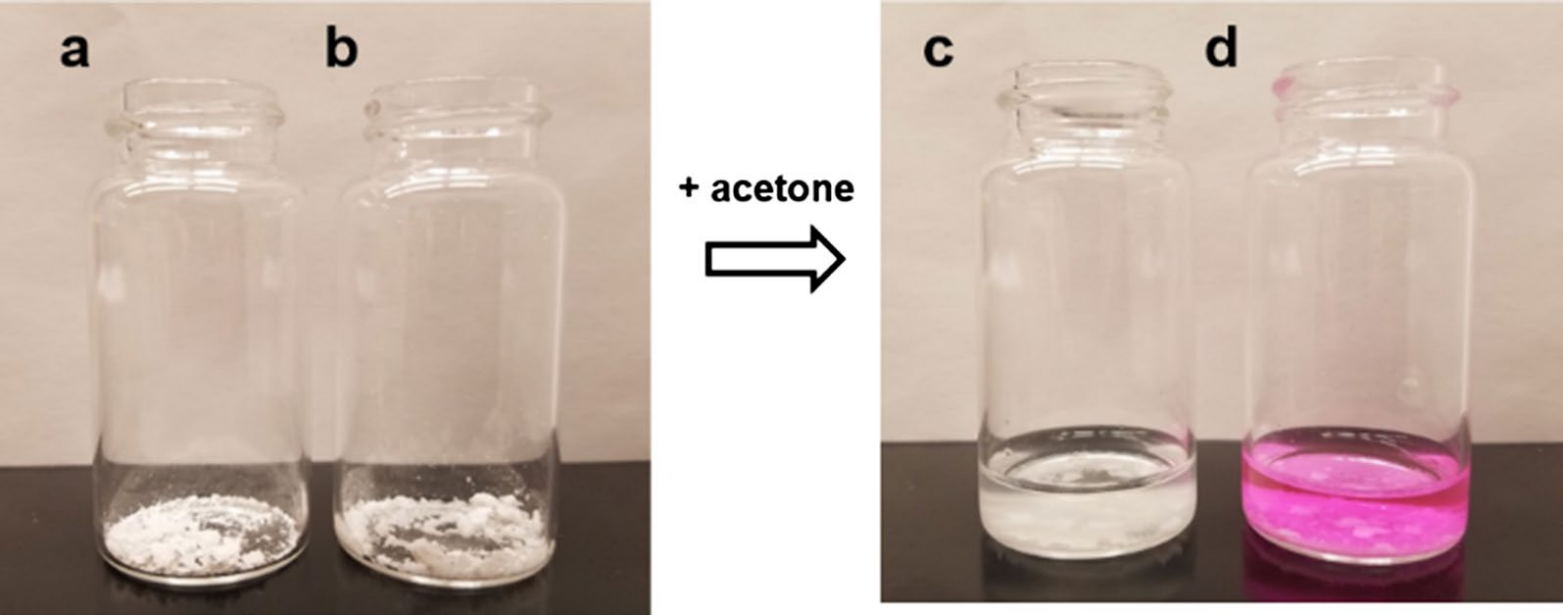
Desorption of Rose Bengal from dry co-polymers samples following exposure to acetone. **a** native dry co-polymer **b** dry polymer following adsorption of Rose Bengal **c** native polymer following exposure to acetone **d** release of Rose Bengal in acetone from the Rose Bengal-complexed copolymer

The chromophore disappearance was surprising, and it was initially hypothesized that the Rose Bengal photobleached in the presence of polymers **1a**–**1l**. Previous reports proposed that aggregation of Rose Bengal or other dyes onto the polymer surface promotes photobleaching and is an irreversible process [47]. To test this theory, the polymer previously exposed to the aqueous Rose Bengal solution was isolated, dried, and placed in acetone. Qualitatively, Rose Bengal desorbed from the polymers and the characteristic pink color of the dye reappeared in the solution (Fig. 5). Thus, the Rose Bengal-polymer complex appears to be a reversible process in which complexation drives loss of the pink color.

**Fig. 6:**
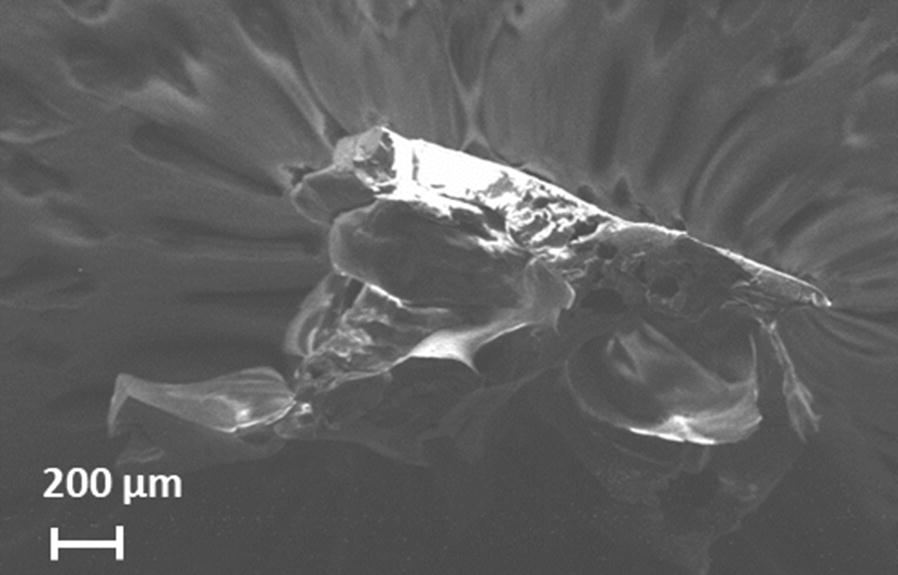
SEM images of triblock copolymer **1l** following treatment of the solid polymer with an aqueous solution of Rose Bengal

Scanning electron microscopy images of the thin film were obtained to further verify the polymer-dye complex (Fig. 6). A smooth Rose Bengal layer appears to blanket itself over the surface of the polymer, which is in contrast to the sharp crevices of the polymer. This smooth surface was not present before exposure to the dye, further providing evidence for the dye being adsorbed onto the polymer.

**Fig. 7:**
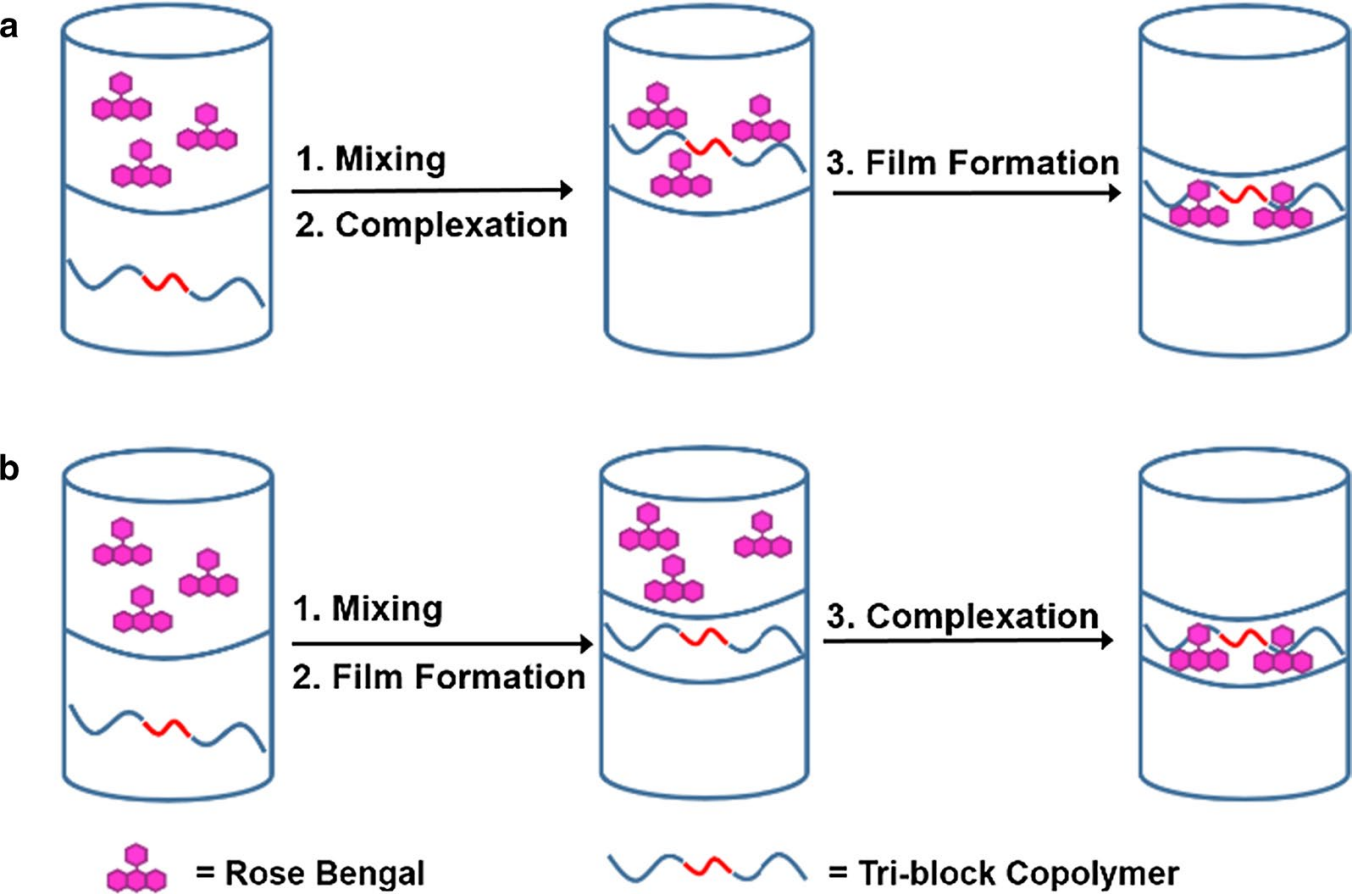
Models to explain the complete removal of Rose Bengal from an aqueous solution using polymer **1**. A film forms between the aqueous and organic phases

Two models were developed to explain the film formed during liquid–liquid extraction of Rose Bengal. The first model involves the impact of polymer solubility before and after complexation with Rose Bengal. Prior to complexation, polymer **1** is soluble only in the organic phase. After mixing the polymer with the Rose Bengal aqueous solution, the dye adsorbs to the polymer. The resulting Rose Bengal-polymer complex is well-hydrated but insoluble in the aqueous and organic phases. A thin film containing both the dye-polymer complex forms between the two layers (Fig. 7a) [47]. In the alternative model, the solubility of polymer **1** alters when exposed to the aqueous solution but before interacting with the Rose Bengal. Upon contact with the aqueous layer, the polymer becomes hydrated. This hydrated polymer is insoluble in the organic layer but is not soluble enough in the aqueous layer. A thin polymer film forms between the two layers. This film is in continuous contact with the aqueous Rose Bengal solution, allowing for the dye to adsorb onto the polymer through a surface complexation mechanism, similar the PLA–PEG nanoparticles designed by Langer (Fig. 7b) [18]. Whichever model is applicable, the removal of small molecule organic pollutants from water mediated by **1** is intricate. Initial studies involving Rose Bengal removal from water were instructive in showing that (1) a small molecule organic pollutant can be removed from water by polymer **1** and (2) the formation of solid Rose Bengal-polymer complex appears to be a favorable endpoint.

**Fig. 8:**
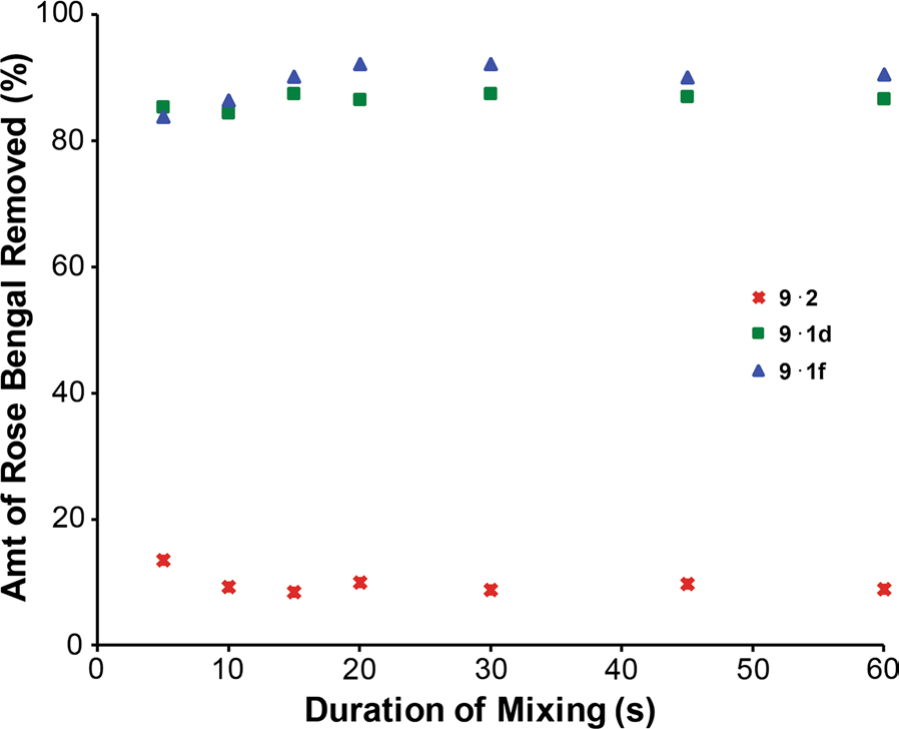
Time course for the removal of Rose Bengal from water mediated by polymers **1d**, **1f**, and **2**

The Rose Bengal removal rate was investigated to determine whether varying the l-lactide to ε-caprolactone ratio influenced dye adsorption. Aqueous Rose Bengal solutions were mixed with dichloromethane solutions of **1d** and **1f** at select time intervals up to 60 s. Aliquots of the aqueous layer were analyzed to quantify the Rose Bengal UV/Vis absorbance at each time point. Both polymers removed greater than 80% of the dye within 5.0 s of mixing with little decreased adsorption between 5 s and 60 s (Fig. 8). The initial Rose Bengal removal rate was 2 × 10^6^ mol/L s for both **1d** and **1f**, indicating that Rose Bengal removal occurred rapidly irrespective of the l-lactide to ε-caprolactone ratio. Homopolymer **2** did not remove significant amounts of the organic dye in the same time period.

Using the 60 s absorbance time point, equilibrium inclusion constants were calculated for polymers **2**, **1d**, and **1f** (Table 5). The inclusion constant values were large, ranging from 1.0 × 10^5^ to 7.9 × 10^5^ M^−1^, similar in magnitude to the values observed between **1l** and the PAHs. The average adsorption capacity for Rose Bengal with polymer **1** was 6.2 × 10^−7^ mol/g polymer. A binding isotherm for Rose Bengal was not feasible due to spectrophotometric interference with Rose Bengal at the low dye concentrations required.

The ∆G° values for Rose Bengal removal from water were calculated for polymer **1** and homopolymer **2**. All polymers had a negative ∆G° value with only one kcal/mol difference between the homopolymer **2** and copolymers **1d** and **1f**. The apparent difference in absorbance changes observed using **2** compared to the triblock copolymers translates into a moderate thermodynamic difference between the two polymer classes. This result may indicate that ∆G° is dominated by entropic effects stemming from the displacement of large numbers of water molecules from the polymer surface upon adsorption of Rose Bengal. Such an effect would be observed upon adsorption of Rose Bengal by either **2** or copolymers **1**.

**Fig. 9:**
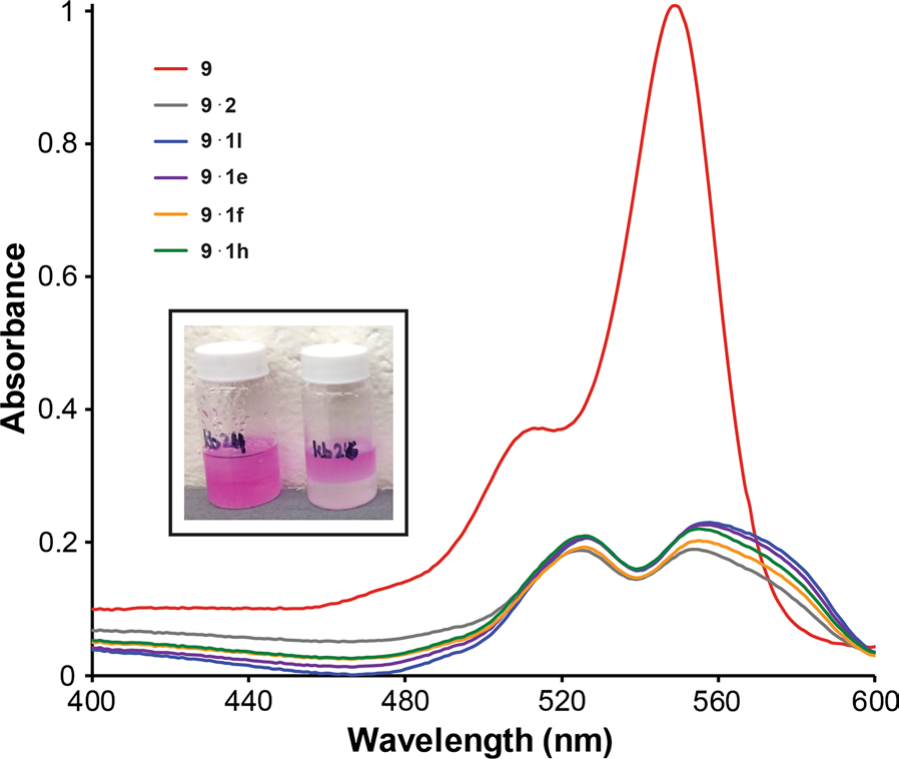
Overlay of UV/Vis spectra obtained during liquid–liquid extraction studies of select aged polymers **1** and 8.3 × 10^−6^ M Rose Bengal. The Rose Bengal absorbance shifted when combined with the polymers. Inset: Comparison of aqueous (top layer) and organic (bottom layer) phases with aged polymer (left vial) and newly formed polymer (right vial)

To examine if polymer shelf-life impacts Rose Bengal removal, polymers **1a**–**1l** were aged for 6 months under ambient laboratory conditions and then utilized in the same extraction studies. A bathochromic shift in the Rose Bengal λ_max_ and a decreased Rose Bengal absorbance signal were noted in the aqueous phase after exposure to the aged polymer (Fig. 9). This is in contrast to the complete disappearance of the Rose Bengal chromophore which was observed for the newly synthesized polymers.

The bathochromic shift suggests that the dye is in a more hydrophobic environment, relative to water. A similar shift has been reported with other macromolecular hosts [41–45]. Based on the Rose Bengal absorbance remaining in the aqueous layer, 80–82% of the dye was removed by the polymers, comparable to the newly synthesized polymers. The aged polymer has different properties compared with the newly formed polymer since the characteristic pink color is present in both layers (Fig. 9).

One theory accounting for the aged polymer results is that the polymer properties change over time, either by polymer decomposition or adsorption of trace impurities. ^1^H NMR spectra of representative aged polymers **1** do not show significant decomposition or other compounds as compared to a similarly newly formed polymer. A comparison of newly formed and aged polymer size exclusion chromatography traces demonstrated that the aged polymer sample degraded into polymeric fragments. Exposure of the organic pollutants to this non-uniform polymer resulted in a complex that had increased solubility in water. A bathochromic shift is observed since the polymer-dye complex would remain in the aqueous layer.

**Fig. 10:**
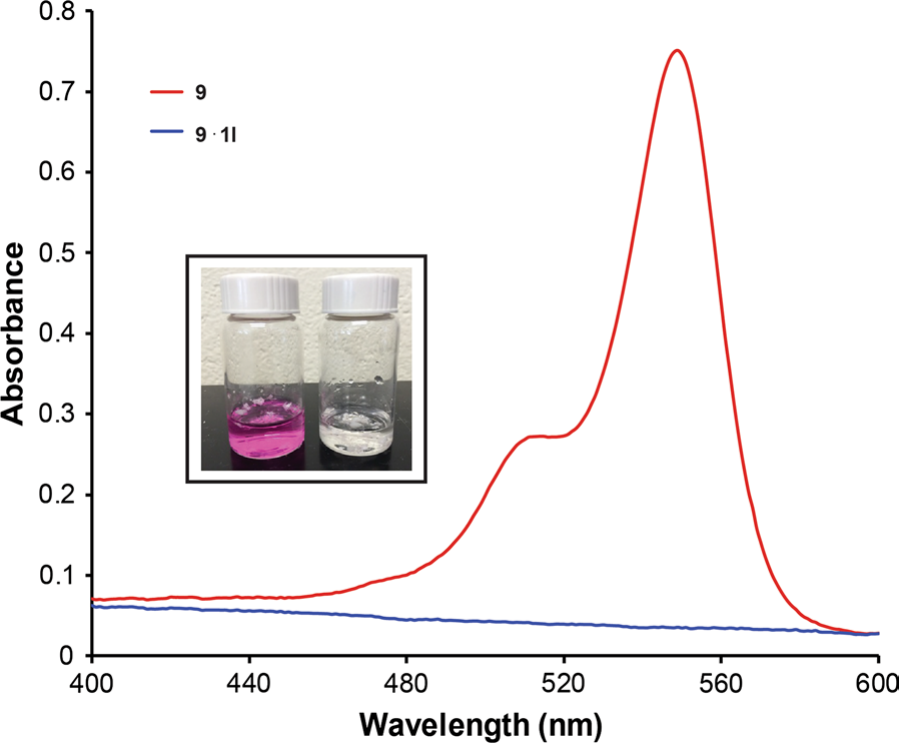
Overlay of Rose Bengal absorbance spectra in an aqueous solution before and after treatment with solid **1l**. Inset: Vials containing an aqueous solution of Rose Bengal and solid **1l** before mixing (left) and after mixing (right). The Rose Bengal color is not present in the aqueous phase following mixing

As a direct comparison to the PAH studies, solid phase complexation studies with aqueous Rose Bengal were performed using newly formed **1l** as a representative triblock copolymer. These studies were executed without an organic solvent, forcing Rose Bengal and the polymer to interact through a surface complexation mechanism. Different aqueous dye solutions were exposed to the polymer and mixed until no pink color was visible in the aqueous layer. Within 10.0 min, the polymer absorbed greater than 90% of the dye as detected by UV/Vis spectroscopy (Fig. 10). The adsorption time increased as the dye concentration grew: while 2.1 × 10^−6^ mmol of polymer **1** can remove 2.1 × 10^−5^ mmol of Rose Bengal within 10 min, it requires 2 h to remove completely 3.3 × 10^−4^ mmol of the dye. Additional time studies were not performed. The results indicate that solid-state adsorption is slower compared to the liquid–liquid extraction method. It is hypothesized that the polymer can remove additional Rose Bengal if given a longer time. In some cases, the solid polymer developed a light pink coating on its exterior, evidence that Rose Bengal adsorbs onto the solid-state polymer surface.

Mechanical properties of poly(lactide) and related copolymers are likely to affect differences in adsorption behaviors. The nature of these differences is strongly dependent on the molecule interaction at the polymer surface. No mechanical studies were performed. However, establishing a baseline correlation between small molecule adsorption and properties such as tensile strength or compression stress would be an important step in large-scale processing and commercialization of sorbent materials based on **1** [48].

Comparison studies were undertaken with the aqueous Rose Bengal solution and 0.050 g of the Brita filter, PEI, PAMAM dendrimer, and β-cyclodextrin, respectively. Brita filters removed an average 4.0% of Rose Bengal from the aqueous solution within 30.0 s as compared with 93% with **1l**. Bathochromic shifts in the Rose Bengal signal from 548 to 553 nm or 561 nm was observed after exposure to the water soluble PEI and the 2nd generation PAMAM dendrimer, respectively. These results suggest that PEI and the PAMAM dendrimer entrap the dye within their interiors and the resulting complexes are soluble in water. The β-cyclodextrin did not remove the Rose Bengal as previously reported [49].

## Conclusion

A series of biorenewable *block*-poly(l-lactide)–*block*-poly(ε-caprolactone)–*block*-poly(l-lactide) triblock copolymers was prepared with varying ratios of **3** and **4**. The copolymers successfully removed phenanthrene over other PAHs from an aqueous environment. Copolymers **1** were more efficient in removing small organic pollutants as compared with traditional removal devices such as Brita filters, PEI, β-cyclodextrin, and PAMAM dendrimers.

Studies demonstrated that polymers **1a** to **1l** removed Rose Bengal from aqueous solution, either through adsorption onto the solid polymer or through solution-phase interactions. While SEM revealed that polymers with different **3**:**4** ratios had varying polymeric morphologies, little impact on the rate or quantity of Rose Bengal removed from water was observed. Removing the hydrophilic block diminished the ability for the polymer to remove organic pollutants from aqueous environments so it is necessary to have the triblock copolymer. Limiting the amount of l-lactide for the polymerization can further decrease the cost of the triblock copolymer and make this system more economically feasible.

The biorenewable triblock copolymers **1** have the prospect for further developing into scaffolds that remove organic pollution from water. Studies directed towards further optimization of the polymer by increasing the hydrophobicity of the internal blocks are currently underway. Internal hydrophobic blocks derived from renewably-sourced monomers are of particular interest. Work to better understand the nature of the solid state complexes formed from these triblock copolymers and small molecule guests and to better predict how organic pollutants interact with the polymers are being addressed and will be reported in due course.

## Materials and methods

### General information

All solvents and reagents were obtained from Sigma-Aldrich or Fisher Scientific and used without further purification. ^1^H NMR spectroscopy studies were conducted using a Bruker AV-300 high performance digital spectrophotometer or a Bruker Avance III HD 400 NMR spectrophotometer. Samples were dissolved in deuterated chloroform (CHCl_3_). ^1^H NMR spectra were referenced to the residual chloroform peak at 7.26 ppm. SEC studies were performed using a Waters Breeze 2 HPLC equipped a differential refractive index (dRI) detector and two Styragel HR 5-μm columns. Tetrahydrofuran (THF) was used as the mobile phase at a flow rate of 0.75 mL/min. SEC data was processed using the Waters Breeze 2 GPC processing software to determine M_w_, M_n_, and PDI values. SEC column calibration employed linear poly(styrene) standards ranging in molecule weight from 1.2 × 10^3^ to 3.7 × 10^5^ Da. SEM studies were conducted using a Zeiss EVO MA10 instrument. Comparative SEC analysis was performed using a Shimadzu Prominence liquid chromatography system equipped with a RID-20A detector and a single Styragel HR 5-µm column. UV–Vis spectroscopy studies were conducted using a Cary 300 Bio Spectrophotometer with Cary2 Win processing software or an Agilent Technologies 845x-UV–Visible System with UV–Visible ChemStation processing software. Fluorescence studies were completed with a Shimadzu RF-5301 PC fluorimeter using RFPC software.

### Preparation of triblock copolymers

A representative synthesis of polymer **1** is described below. Polymers with different block ratios of **3**:**4** were prepared by varying l-lactide.

To a scintillation vial was added 14.0 mg (0.101 mmol) of 1,4-benzenedimethanol (**5**), 1.40 mL (12.3 mmol) of ε-caprolactone (**4**), and 1.23 mL (0.230 mmol) of 1 M HCl in diethyl ether. After stirring at room temperature for 30 min, the vial was wrapped with parafilm and left to stand under ambient conditions. After 48 h, vigorous air flow was used to remove the acid catalyst for 15 min. To the vial was added 1.70 g (11.8 mmol) of l-lactide (**3**) and 0.76 mL (0.53 mmol) of 0.2 M tin (II) octanoate in toluene. The vial was heated at 130 °C for 1 h. The polymer then was cooled to room temperature before being dissolved in warm ethyl acetate and transferred to 50 mL of methanol. The reaction mixture was cooled for 15–30 min to encourage precipitation. The final triblock copolymer product precipitated as a white solid and was recovered by vacuum filtration. This reaction was repeated with varying amounts of **3**, ranging from 0.0 to 11.8 mmol (Table 1) to examine how varying the ratio of **3**:**4** impacted the physical and small molecule complexation properties of the resulting block copolymer. For each combination of **3** and **4**, duplicate or triplicate synthetic trials were performed, and the average isolated yields of the polymers ranged from 68 to 83%. Each polymer was characterized using ^1^H NMR, FT-IR spectroscopy and size-exclusion chromatography (SEC). Characterization was similar to previously reported systems [50].

### Scanning electron microscopy (SEM)

SEM studies were performed by loading carbon tapes with 3.0 to 4.0 mg of the solid polymer sample. SEM images were obtained using a Zeiss EVO MA10 Lab6 scanning electron microscope to observe the morphology and surfaces of the polymers.

### Removal of environmental pollutants from water

#### PAH removal using solid polymer

Saturated aqueous PAH solutions and 0.050 g of a solid polymer sample were mixed for 30 s. The solution was allowed to settle and the fluorescence spectrum of the aqueous supernatant was measured. The fluorescence emission was recorded at the excitation wavelengths for the following PAHs: fluoranthene, 255 nm; phenanthrene, 351 nm; and pyrene, 334 nm. The percent removal of each PAH was determined by calculating the % decrease in fluorescence using Eq. 1, where *I* is the fluorescence emission intensity at the recorded wavelength specific to each PAH.

1 $$\% \; Removal\;PAH = 100 \times \frac{{\left( {I_{initial} - I_{complex} } \right)}}{{I_{initial} }}$$

#### Rose Bengal removal using polymer dissolved in dichloromethane

Each polymer was dissolved with dichloromethane in a volumetric flask to give a concentration of 1.00 × 10^−4^ M. Rose Bengal was dissolved in deionized water to produce an 8.3 × 10^−6^ M solution. A 20 mL glass scintillation vial was charged with 5.0 mL of the organic polymer solution and 5.0 mL of the dye solution. The vial was mixed for 30 s. UV–Vis spectroscopy was used to measure the absorbance spectrum from 400 to 600 nm of the aqueous phase. The percent removal of Rose Bengal from the aqueous phase was determined by calculating the percent decrease in absorbance at 549 nm (Eq. 2).

2 $$\% \; Removal\;Rose\;Bengal = 100 \times \frac{{\left( {A_{initial} - A_{complex} } \right)}}{{A_{initial} }}$$

#### Varying Rose Bengal concentrations when exposed to solid polymer

To a 20 mL scintillation vial was added 5 mL of an 8.3 × 10^−6^ M aqueous solution of Rose Bengal to 0.050 g of solid polymer. The vial was kept in the dark and stirred until the solution was colorless. The mixture was allowed to settle and the UV/Vis spectrum of an aliquot of the aqueous supernatant was recorded. The percent Rose Bengal removed from the aqueous phase as determined using Eq. 2.

#### Calculation of inclusion constants and adsorption capacity

The following equilibrium equation was used to determine the inclusion formation constants (*K*_*i*_) and the corresponding Gibbs free energy (ΔG°) values [51].

$${\text{polymer}} + {\text{guest}} \to {\text{polymer{-}guest}}$$

3 $$K_{i} = \left[ {\text{polymer{-}guest}} \right]/\left( {\left[ {\text{polymer}} \right]\left[ {\text{guest}} \right]} \right)$$

[polymer-guest] is the concentration of the polymer with a PAH or Rose Bengal, [polymer] is the concentration of the polymer prior to exposure to PAH or Rose Bengal, [guest] is the concentration of PAH or Rose Bengal in the aqueous solution after equilibration with the polymer after 60 s. The [guest] value used for the calculations corresponded to the concentration when maximum guest adsorption was observed.

It was assumed that the polymer and the polymer-guest complex exist in the solid state after equilibrium as they appear to precipitate as a thin film. Therefore, their concentration values were taken to be 1. The guest concentrations were calculated using the absorbance or fluorescence emission maxima at 549 nm and calibration curved for each guest (see Additional file 1). Based on the above results, the inclusion formation constant was simplified to *K*_*i*_ = 1/[guest] [37]. The Gibbs free energy was determined using the following equation

4 $$\Delta {\text{G}}^\circ = - {\text{RTln}}K_{i}$$

where *K*_*i*_ is the inclusion formation constant.

Adsorption capacity values were obtained by using the calibration plot shown in Additional file 1 to determine the molar concentration of each PAH or Rose Bengal before and after exposure to the polymer. The amount used after exposure to the polymer corresponded to the maximum amount of guest removed under equilibrium conditions, as determined spectroscopically. The amount of guest (in moles) divided by the mass of polymer (0.050 g) in each experiment was calculated to provide the adsorption capacity according to Eq. 5.

5 $$Adsorption \;Capacity = V \left( L \right) \times \frac{{\left( {C_{initial} - C_{final} } \right)}}{S}$$

where *V* is the volume of guest solution exposed to the polymer, *C*_*initial*_ *and C*_*final*_ are the molar concentrations of guest in solution before and after exposure to the polymer, and *S* is the mass of polymer in grams.

## Supplementary information

**Additional file 1.** Experimental data for the characterization of polymers **1** and the PAH and Rose Bengal spectroscopy studies.

## Data Availability

The dataset supporting the conclusion of this article is included within the article and its additional files.

## Abbreviations

BPA: bis-phenol A
CHCl_3_: chloroform
dRI: differential refractive index
*K*_*i*_: inclusion constant value
PAH: polycyclic aromatic hydrocarbon
PAMAM: poly(amidoamine)
PDI: polydispersity index
PEG: poly(ethylene glycol)
PEI: poly(ethylene imine)
PLLA–PCL–PLLA: *block*-poly(l-lactide)–*block*-poly(ε-caprolactone)–*block*-poly(l-lactide)
POPs: persistent organic pollutants
RSD: relative standard deviation
SEC: size-exclusion chromatography
SEM: scanning electron microscopy
THF: tetrahydrofuran
