# Supplementary material for: Biorenewable triblock copolymers consisting of l-lactide and ε-caprolactone for removing organic pollutants from water: a lifecycle neutral solution
Source: BMC Chem. 2019 Oct 22;13(1):122. doi: 10.1186/s13065-019-0638-z (PMC6805438; doi:10.1186/s13065-019-0638-z)
Supplement: Supplementary file 1 — Additional file 1. Experimental data for the characterization of polymers 1 and the PAH and Rose Bengal spectroscopy studies. [file 13065_2019_638_MOESM1_ESM.docx]

Biorenewable Triblock Copolymers Consisting of L-Lactide and ε-Caprolactone for Removing Organic Pollutants from Water: A Lifecycle Neutral Solution

Katrina T. Bernhardt,^†^ Haley G. Collins, ^‡^ Amy M. Balija^‡*^

^†^Fordham University, Department of Chemistry, 441 East Fordham Road, Bronx NY, 10458

^‡^Radford University, Department of Chemistry, P.O. Box 6949, Radford, VA 24142

*Corresponding Author: abalija@radford.edu

**ADDITIONAL INFORMATION**

**Table of Contents**

| **Section** | **Topic** | **Page(s)** |
| --- | --- | --- |
| I. | Synthetic scheme of tri-block copolymers **1a**-**1l** | 2 |
| II. | ^1^H NMR characterization of polymers **1a**-**1l** | 3-4 |
| III. | Calculating polymer M_n_ values | 5 |
| IV | *E* values | 5 |
| V. | FT-IR characterization of tri-block copolymers | 6 |
| VI. | Size exclusion chromatography (SEC) | 6-7 |
| VII. | Fluorescence spectroscopy studies | 7-12 |
| VIII. | UV/Vis spectroscopy studies of liquid-liquid extraction | 12-14 |
| IX. | References | 15 |

**I.** **Synthetic scheme of tri-block copolymers 1a-1l**

**II. ^1^H NMR characterization of polymers 1a-1l**

A representative ^1^H NMR spectrum of tri-block copolymer **1a**-**1l** is shown below along with ^1^H NMR spectra of poly(ε-caprolactone) (**2**) and poly(L-lactide). Peak assignments in the ^1^H NMR spectra were confirmed by comparing spectra representing **1a**-**1l**, poly(L-lactide), and 1,4-benzenedimethanol (**5**). The distinct peak at 7.33 ppm was assigned to H1. Peaks at 4.05 ppm (H7), 2.30 ppm (H3), 1.64 ppm (H4 and H6), and 1.37 ppm (H5) were present in Spectra 2a and 2c (Fig. S1), but not 2b, and their respective integration ratio is 1:1:2:1, supporting the assignment of these hydrogen peaks in the poly(ε-caprolactone) region. Similarly, the peaks at 5.15 ppm (H8) and 1.55 ppm (H9) are present in Spectra 2b and 2c (Figure SI_1) but not in 2a, and their respective integration ratio is 1:3, supporting the assignment to the hydrogens in the poly(L-lactide) region.

**a.**

**Fig. S1** a.) Structure of a *block*-poly(L-lactide)-*block*-poly(ε-caprolactone)-*block*-poly(L-lactide) highlighting diagnostic protons used in ^1^H NMR analysis. A comparison of ^1^H NMR spectra is shown for for b.) poly(ε-caprolactone) **2**, c.) poly(L-lactide), and d.) a representative triblock copolymer of structure type **1**

**b.**


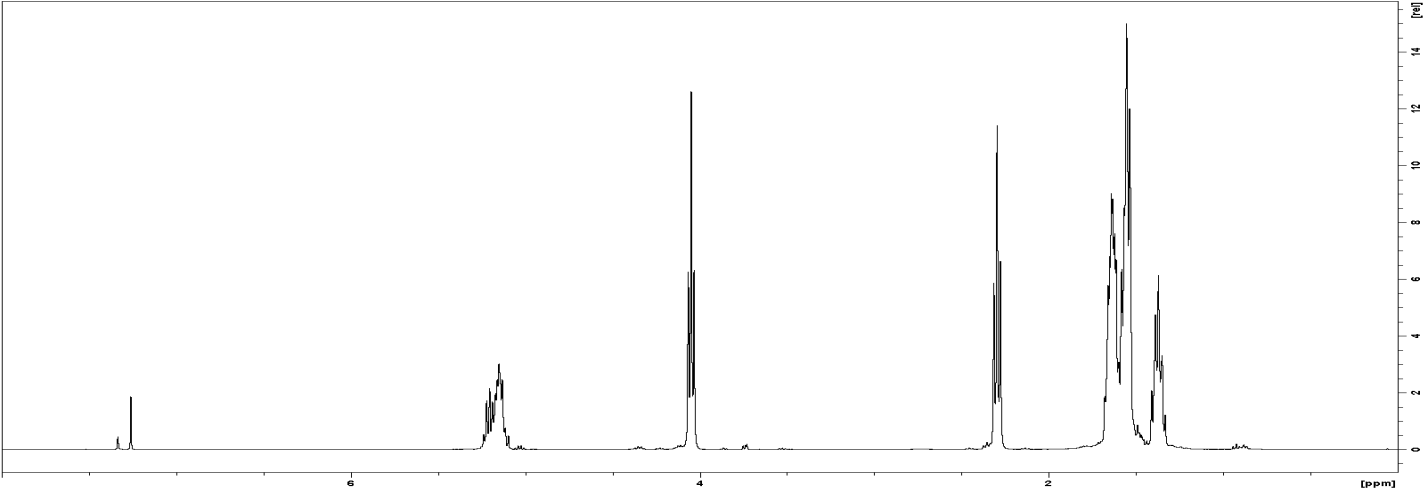

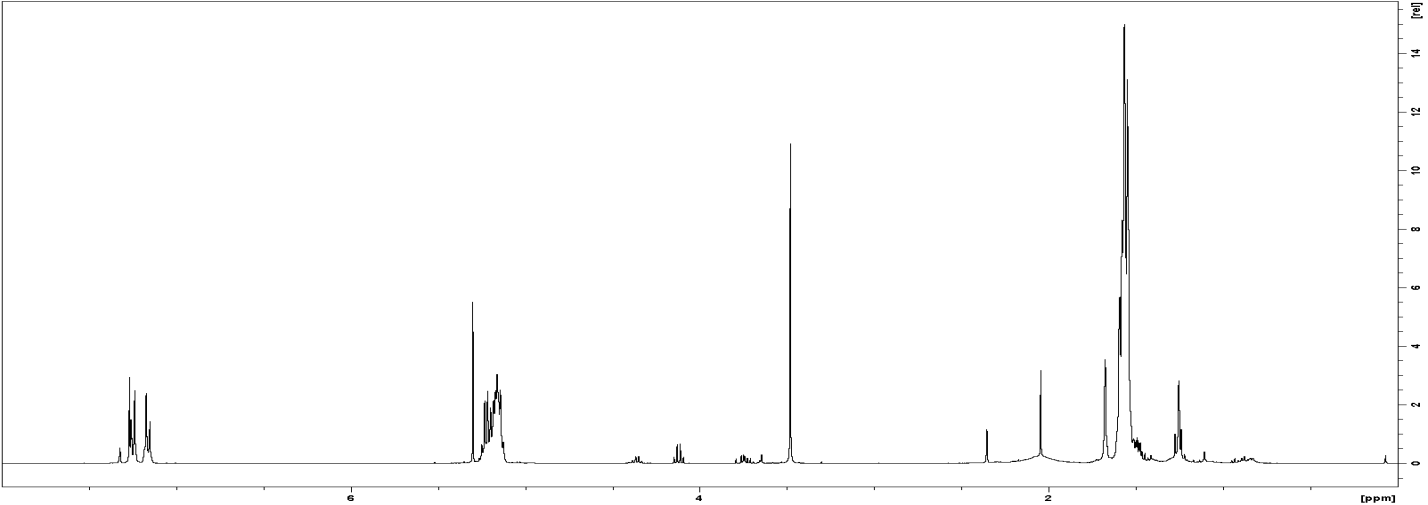

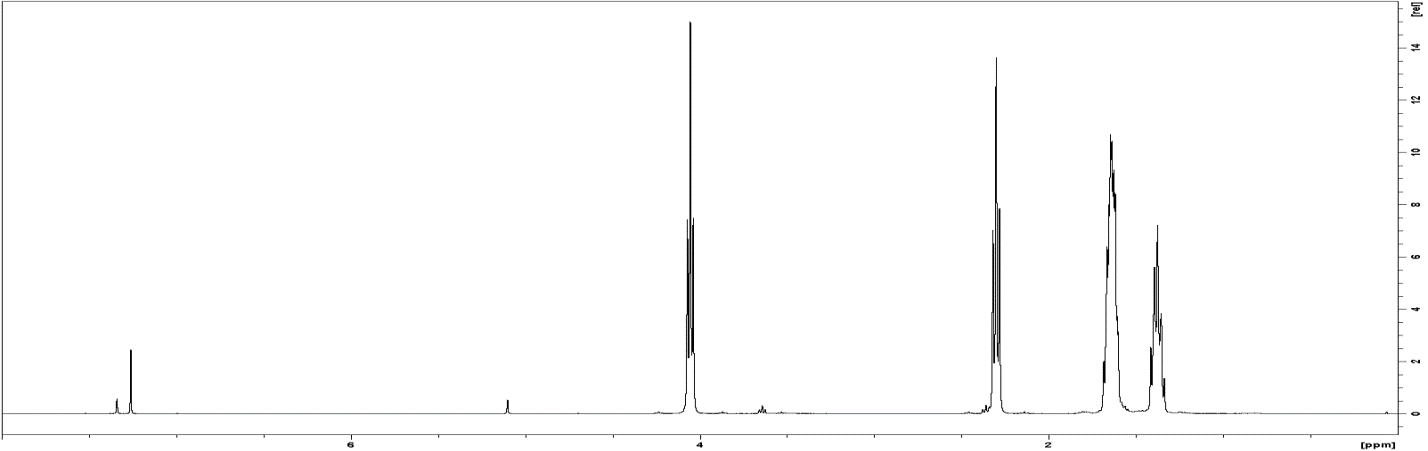


8 ppm 7 6 5 4 3 2 1 0

**c.**

**d.**

**III. Calculating Polymer M_n_ Values**

The relative hydrogen peak integrations were utilized to calculate the polymer experimental molecular weights. In all the reactions, 12.3 mmol of ε-caprolactone was used. The relative integrations of the poly(ε-caprolactone) hydrogen peaks were consistent, indicating approximately 180 ε-caprolactone units per polymer. Proportional changes in the poly(L-lactide) hydrogen peak integrations were observed in the ^1^H NMR spectrum as the amount of **3** varied. The incremental increases in number of L-lactide units were evident in the calculated polymer molecular weights. Shown in Fig. S2 is the linear relationship between addition of **3** and polymer molecular weight.

**Fig. S2** Relationship between M_n_ the mole ratio of L-lactide (**3**) and ε-caprolactone (**4**)

**IV. *E* Values**

*E* values were calculated by determining the quotient of the combined waste mass produced divided by the mass of the isolated product in each polymerization reaction. In this work, the combined waste mass was the sum of solvents used, unrecovered catalysts, and unrecovered starting materials. The *E* values reported in Table 1 of the article were averaged over two or three replicated polymerization trials.

**V.** **FT-IR characterization of polymers 1a-1l**

FT-IR spectroscopy studies were performed using a Thermo Scientific iS50 FT-IR spectrophotometer. FT-IR spectra were obtained for the polymers. Characteristic peaks near 1700 cm^-1^ and 3000 cm^-1^ represent the C=O and C-H bonds, respectively.

**VI. Size Exclusion Chromatography (SEC)**


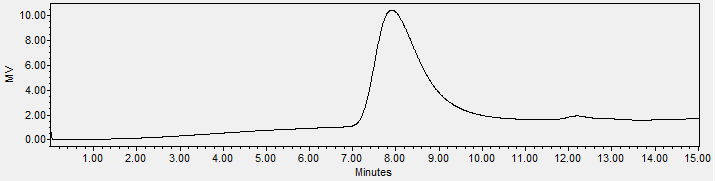
A representative size-exclusion chromatograph is shown below for polymer **1g**. Chromatographs for ε-caprolactone (**4**) and 1,4-benzenedimethanol (**5**) are shown for comparison.

**a.**

**b.**


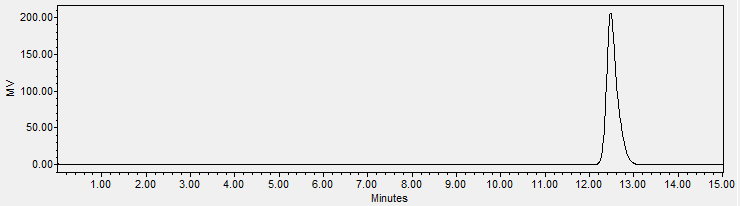


**
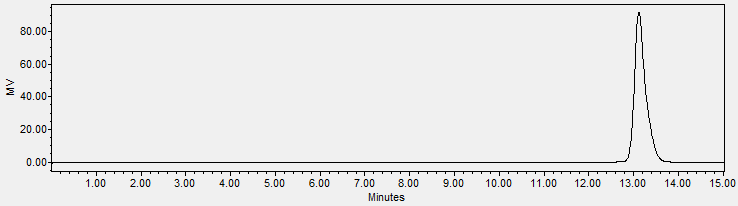
**

**c.**

**Fig. S3** SEC graphs for a.) *block*-poly(L-lactide)-*block*-poly(ε-caprolactone)-*block*-poly(L-lactide) (**1g**), b.) 1,4-benzenedimethanol (**5**), and c.) ε-caprolactone (**4**)

A representative size-exclusion chromatograph is shown below for newly prepared polymer **1g** (blue) compared with an aged polymer **1g** (black) in Fig. S4. Over time, the polymer degrades into smaller polymeric fragments.

**Fig. S4** SEC graphs for *block*-poly(L-lactide)-*block*-poly(ε-caprolactone)-*block*-poly(L-lactide) (**1g**) newly prepared (blue) and aged (black)

SEC column calibration employed linear poly(styrene) standards ranging from 1.2 x 10^3^ Da to 3.7 x 10^5^ Da. The M_n_ and M_w_ values determined by SEC were 14-to 28-fold lower than the NMR-derived M_n_ values. This result is in contrast to previous reports suggesting that linear poly(styrene) calibration standards overestimate the molar mass of PLA containing polymers.^1,2^ The lower M_n_ and M_w_ values for **1** could result from **1** adopting a solution conformation different than linear poly(styrene), giving differences in the hydrodynamic volumes of the two systems. Alternatively, the lower M_n_ and M_w_ values could be due to error in dRI in determining molecular weights for this polymer class. Other detection methods, such as multi-angle light scattering, could give more accurate M_n_ and M_w_ values.^3^

1. **Fluorescence Spectroscopy Studies**

**Fig. S5** Structures of polycyclic aromatic hydrocarbons (PAHs) studied

Dichloromethane stock solutions of each PAH were prepared according to the table below using a 10 mL volumetric flask.

**Table S1** Preparation of PAH stock solutions from dichloromethane

| **PAH** | **Amt (mg)** | **Final Volume** |
| --- | --- | --- |
| Pyrene | 75 | 10 mL |
| Fluoranthene | 75 | 10 mL |
| Phenanthrene | 66 | 10 mL |

Saturated aqueous solutions of each PAH were prepared by adding the amounts listed in Table S2 of the dichloromethane stock solution to the appropriate volumetric flask. The dichloromethane was evaporated by a stream of compressed air. Deionized water was added to the volumetric flask, filling to the calibration mark. A suspension was obtained which was then sonicated to ensure thorough mixing.

**Table S2** Preparation of saturated aqueous PAH solutions for fluorescence studies

| **PAH** | **Volume of Dichloromethane Stock Solution (mL)** | **Final Volume** |
| --- | --- | --- |
| Pyrene | 1.0 | 250 mL |
| Fluoranthene | 1.0 | 250 mL |
| Phenanthrene | 0.4 | 100 mL |

Control studies were completed with the standard stock solution. Approximately 4 mL of each saturated PAH solution was filtered through a cotton plug into a quartz cuvette. Using the Shimadzu RF-5301 PC spectrofluorophotometer, fluorescence emission spectra were obtained using the instrumental parameters described below in Table S3. Each PAH stock solution was tested in triplicate to ensure that consistent fluorescence spectra were obtained.

**Table S3** Instrument parameters used for fluorescence spectroscopy studies

| **Parameter** | **Value** |
| --- | --- |
| Excitation slit | 10 nm |
| Emission slit | 10 nm |
| Sensitivity | Low |
| Response | Auto |
| Scan Speed | Super |
| Scan range | 352 nm to 800 nm |
| Excitation Wavelengths | Fluoranthene: 255 nm Phenanthrene: 351 nm  Pyrene: 334 nm |

Solid-phase removal studies were prepared by adding 50.0 mg portions of copolymers **1** into 20 mL scintillation vials. A 5.0 mL aliquot of a filtered saturated PAH solution from above was added to the scintillation vial. The vial was capped and shaken for 30 s. The mixture was allowed to settle and an aqueous aliquot was filtered through a cotton plug into a quartz cuvette. Fluorescence spectra of the PAH solution were obtained using the instrument parameters in Table S3. The percent removal of each PAH was determined by calculating the % decrease in fluorescence using equation 1, where *I* is the fluorescence emission intensity at the recorded wavelength specific to each PAH.

| $\% Removal PAH=100 \times\frac{(I_{initial}-I_{complex})}{I_{initial}}$ | (1) |
| --- | --- |

Calibration curves for the three PAH examples were generated using fluorescence spectroscopy to calculate the values of K*_i_* and adsorption capacity for each compound studied (Figure SI_6 through Figure SI_8). A 5-mL portion of each calibration solution was then introduced to 50 mg of polymer **1l**. A decrease in fluorescence intensity for each PAH at every concentration level was observed in the presence of the polymer. The change in fluorescence intensity (ΔI) was plotted vs. PAH concentration to generate binding isotherms for each analyte.

**Fig. S6** Concentration dependence of pyrene emission at 394 nm (top) and adsorption isotherm for pyrene on polymer **1l** (bottom)

**Fig. S7** Concentration dependence of fluoranthene fluorescence emission at 387 nm (top) and adsorption isotherm for fluoranthene on polymer **1l** (bottom)

**Fig. S8** Concentration dependence of phenanthrene fluorescence emission at 387 nm (top) and adsorption isotherm for phenanthrene on polymer **1l** (bottom)

1. **UV-Vis Spectroscopy Studies of Liquid-Liquid Extraction**

UV/Vis spectroscopy studies were conducted using a Cary 300 Bio spectrophotometer with Cary2 Win processing software. Spectra were collected from 400-600 nm. The percent removal of Rose Bengal was calculated as the change in absorbance at 549 nm (Equation 1) where A is absorbance values.

| $\% Removal Rose Bengal=100 \times\frac{(A_{initial}-A_{complex})}{A_{initial}}$ | (2) |
| --- | --- |

Table S4 compares the average percent removal of Rose Bengal from water across polymers **1a-1l** and for the homopolymer **2** over three trials.

**Table S4** Comparison of % Removal of Rose Bengal from water after 30 s using polymers **1a**-**1l**

| **Polymer** | **L-lactide: ε-caprolactone**  **mole ratio** | **% Removal after 30 s** |
| --- | --- | --- |
| **1a** | 0.056 | 91% |
| **1b** | 0.11 | 83% |
| **1c** | 0.17 | 92% |
| **1d** | 0.23 | 91% |
| **1e** | 0.28 | 92% |
| **1f** | 0.34 | 94% |
| **1g** | 0.45 | 84% |
| **1h** | 0.56 | 93% |
| **1i** | 0.68 | 90% |
| **1j** | 0.79 | 51% |
| **1k** | 0.90 | 86% |
| **1l** | 0.96 | 94% |
| **2** | 0.0 | 33% |

Full UV/Vis spectra were obtained for time points between 5 and 60 s in the time course Rose Bengal removal studies by polymers **1d** and **1f**. A sharp drop off in the Rose Bengal absorbance occurred after 5 s, indicating that an equilibrium was established by 60 s after the start of mixing.

A standard calibration plot of the absorbance at 549 nm was obtained to quantify the amount of Rose Bengal in water (Fig. S6). The values from the 60 s time points were used to calculate the equilibrium concentration of Rose Bengal in mol/L.

**Fig. S9** Concentration dependence of Rose Bengal absorbance at 549 nm

Solid-liquid extractions between polymer **1** and Rose Bengal were done to examine the time necessary for the polymer to remove all the Rose Bengal based on dye concentration. The polymer was slower to adsorb the dye after 1.5 mmol of Rose Bengal (Fig. S10).

**Fig. S10** Relationship between mixing time and the amount of Rose Bengal adsorbed by solid co-polymer **1l**

1. **References**

(1) Kowalski A, Duda A, Penczek S. Polymerization of l,l-lactide initiated by aluminum isopropoxide trimer or tetramer. Macromolecules. 1998;31:2114-2122.

(2) Ma H, Okuda J. Kinetics and mechanism of l-lactide polymerization by rare earth metal silylamido complexes:  effect of alcohol addition. Macromolecules. 2005;38:2665-2673.

(3) Martello MT, Hillmyer, MA. Polylactide–poly(6-methyl-ε-caprolactone)–polylactide thermoplastic elastomers. Macromolecules. 2011;44:8537–8545.
